# The Employment of the Waiting Years

**Published:** 1918-05-04

**Authors:** 


### THE EDITOR'S LETTER-BOX.

### The Employment of the Waiting Years.

### IS PREVIOUS TRAINING DETRIMENTAL?

*To the Editor of* The Hospital.

Sir,—In last week's issue of The Hospital "A Teaching Sister-in-Charge," writing on the above subject, deals with a most interesting and, to my mind, a most important subject. Her ideas on advising the girls at school as to their future, and especially with regard to a nursing career, are excellent and practical.

I cannot, however, agree with her as to the objection to previous training before the general training. It is too sweeping a statement to say almost any scientific or literary subject would be good (that is, in regard to filling in waiting years), except anatomy, physiology, or nursing. These only lead to confusion of mind and staleness later on. For the same reason I deprecate training of any kind in sick nursing before the general training.

I quite agree that this may apply to some girls who are not gifted with enough common sense to recognise that the previous training, though valuable, does not justify them in beginning their general training with the spirit of "Oh! you need not try to teach me anything. I have been in hospital before!" I have met such, but I have also met and had under my charge others who have had training in a special hospital, and who in coming for general training have not only begun in the right spirit, but have proved themselves most capable, and have made excellent nurses in the truest sense of the term.

As matron of a special hospital from which nurses pass on to large general hospitals for training, I feel I must protest against the idea of previous training being detrimental to a nurse's general training.

I find that the matrons of the large training-schools are glad to take probationers whom I thoroughly recommend.—I am, yours faithfully,

April 29, 1918. Member of College of Nursing.

### The Site of St. Mark's Hospital.

*To the Editor of* The Hospital.

